# Relationships of Fat and Muscle Mass with Chronic Kidney Disease in Older Adults: A Cross-Sectional Pilot Study

**DOI:** 10.3390/ijerph17239124

**Published:** 2020-12-07

**Authors:** Bokun Kim, Hyuntae Park, Gwonmin Kim, Tomonori Isobe, Takeji Sakae, Sechang Oh

**Affiliations:** 1 Department of Sports Health Care, Inje University, Gimhae 50834, Korea; fabulousbobo79@gmail.com; 2 Faculty of Medicine, University of Tsukuba, Ibaraki 305-8575, Japan; tiso@md.tsukuba.ac.jp (T.I.); takejisakae@gmail.com (T.S.); 3 Department of Health Science, Dong-A University, Pusan 49315, Korea; 4 Health Convergence Medicine Laboratory, Biomedical Research Institute, Pusan National University Hospital, Pusan 49241, Korea; rlarnjsals47@gmail.com

**Keywords:** chronic kidney disease, fat mass, muscle mass

## Abstract

This cross-sectional pilot study aimed to assess the relationships of fat and muscle mass with chronic kidney disease (CKD) in older adults. Serum creatinine concentration was used to measure estimated glomerular filtration rate (mL/min/1.73 m^2^) in the 236 subjects, who were allocated to three groups: a normal (≥60.0), a mild CKD (45.0–59.9), and a moderate to severe CKD (<45.0) group. The Jonckheere-Terpstra test and multivariate logistic regression were employed to assess body composition trends and the relationships of % fat mass (FM) or % muscle mass index (MMI) with moderate-to-severe CKD. Body weight, fat-free mass, MMI, and %MMI tended to decrease with an increase in the severity of CKD, but the opposite trend was identified for %FM. No relationship with BMI was identified. The participants in the middle-high and highest quartile for %FM were 6.55 and 14.31 times more likely to have moderate to severe CKD. Conversely, the participants in the highest quartile for %MMI were 0.07 times less likely to have moderate to severe CKD. Thus, high fat and low muscle mass may be more strongly associated with CKD than obesity per se.

## 1. Introduction

Chronic kidney disease (CKD) is increasingly recognized as a major public health problem [1,2,3]. The prevalence of kidney disease in the older population in Korea is 31%, according to the Korean National Health and Nutrition Examination Survey [4]. It has been reported for the last two decades that the prevalence of kidney disease is growing rapidly among older adults, and the increase in the prevalence of kidney disease has paralleled the increase in the prevalence of obesity [1,5,6,7]. Thus, on the basis of previous reports, aging and obesity have been recognized as causes of CKD.

Body mass index (BMI) is widely calculated for the assessment of obesity, but it is a composite measure that does not distinguish the independent influences of fat and muscle mass [8,9]. Body composition changes with age, with a loss of muscle mass and the accumulation of fat mass [8,10] resulting in a net weight loss in the older population. Owing to this aging process, many older adults demonstrate a higher percentage whole-body fat mass (FM) and a lower percentage muscle mass, but non-obesity on the basis of BMI [8]. In recent years, it has become gradually accepted that such sarcopenic obesity confers greater susceptibility than obesity defined using BMI to several medical conditions, including diabetes, cardiovascular complications and cerebrovascular disease [11]. Accordingly, the assessment of muscle and fat mass may be a better measurement than BMI for the management of the health and diseases of older adults.

To date, however, the relationship between body composition and CKD has not been thoroughly investigated in older adults. The lack of research on this issue reflects a failure to appreciate the importance of body composition in older adults [12]. Therefore, in this cross-sectional pilot study, we assessed the relationships of FM and muscle mass with CKD in older adults.

## 2. Materials and Methods 

### 2.1. Participants

**Figure 1:**
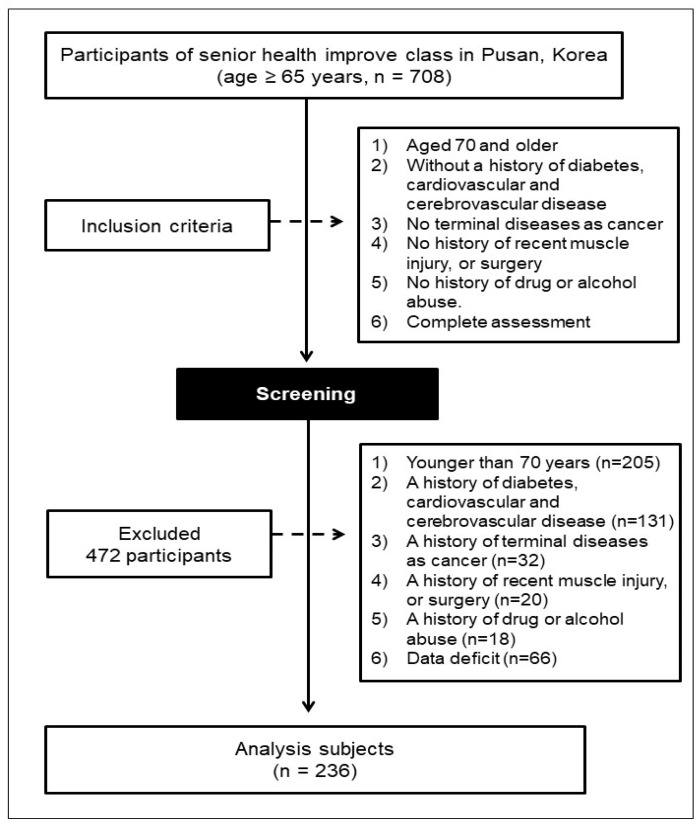
Flow diagram of the enrollment and classification of the study participants.

As shown in Figure 1, the participants in the present study were selected from among the 708 participants in a Senior health improvement class that was held during 2017 and 2018 in health centers for community-dwelling adults aged ≥ 65 year, in Pusan, Korea. The inclusion criteria were: (1) age ≥ 70 year; (2) no history of diabetes, or cardiovascular or cerebrovascular disease; (3) no terminal disease, such as cancer; (4) no history of recent muscle injury or surgery; and (5) no history of drug or alcohol abuse. On the basis of these inclusion criteria, 472 individuals were excluded: 205 who were < 70 years old (*n* = 205); 131 who had a history of diabetes, or cardiovascular or cerebrovascular disease; 32 who had a history of cancer; 20 who had had recent muscle injury or surgery; 18 who had a history of drug or alcohol abuse; and 66 for whom data were missing. Therefore, 236 older Koreans (47 men and 189 women) were included in the study. All the participants provided their informed written consent, and the study was carried out in accordance with the principles of the Declaration of Helsinki and was approved by the Dong A institutional review board (IRB) of the Dong-A University, Korea (No. BR-009-15). 

### 2.2. Anthropometry and Body Composition

We evaluated standing height to the nearest 0.1 cm using a wall-mounted stadiometer. Body weight was measured to within 0.1 kg with a digital electronic scale, with each participant in a light gown. Using this information, BMI was calculated as weight (kg)/height (m^2^). body composition data were evaluated by using multifrequency bioelectrical impedance (InBody S10, Biospace Co. Ltd., Seoul, Korea), which yielded the whole-body lean mass (LM), appendicular skeletal muscle mass (ASM), and percentage (%) FM. To generate the muscle mass index (MMI), we calculated a height-adjusted index by appendicular muscle mass (kg)/height (m^2^) [13,14]. The %MMI was calculated by appendicular muscle mass (kg)/body weight, and multiplying the result by 100% [13,14]. Table S1 indicates the average values of estimated glomerular filtration rate eGFR and body composition parameters according to body composition quartile.

### 2.3. Estimated Glomerular Filtration rate (eGFR) and Biochemical Measurements

Systolic (SBP) and diastolic (DBP) blood pressures were measured on the right arm by using an electronic sphygmomanometer. Blood samples were collected the morning after ≥ 8 h fast statues. Serum levels of total cholesterol (TC), triglycerides (TG) and creatinine were analyzed enzymatically, and high-density lipoprotein-cholesterol (HDLC) was analyzed by the heparin-manganese precipitation method. Levels of fasting plasma glucose (FPG) and insulin were analyzed by the glucose oxidase method and radioimmunoassay, respectively. eGFR was calculated using the new Japanese coefficient-modified Modification of Diet in Renal Disease study equation: eGFR (mL/min/1.73 m^2^) = 194 × (serum creatinine) − 1.094 × (age) − 0.287 (× 0.739 for women) [15]. The participants were allocated to three groups according to eGFR tertiles: a normal (≥60.0 mL/min/1.73 m^2^) group, a mild CKD (45.0–59.9 mL/min/1.73 m^2^) group, and a moderate-to-severe CKD (<45.0 mL/min/1.73 m^2^) group [15,16].

### 2.4. Statistical Analysis

Data were presented as means ± standard deviation or % (number of cases) in Table 1, and means and 95% CI in Table 2, Table 3 and Table 4. Differences between men and women were analyzed using the χ-square test for categorical data and the unpaired *t*-test for continuous data. One-way analysis of covariance (ANCOVA), with age and sex as covariates, was used to compare mean anthropometric, body composition, and biochemical characteristics among the three groups; except for age, which was analyzed using one-way analysis of variance (ANOVA). The Bonferroni post-hoc test was then used when the ANOVA or ANCOVA scores showed significant differences (*p* < 0.05). For data that were not normally distributed, the Kruskal-Wallis test was used to analyze differences among the groups (*p* < 0.05). The Jonckheere-Terpstra test was used to determine the trends in the values in the three groups (two-tailed, with a statistical significance of *p* < 0.05). The Jonckheere-Terpstra test generates a standardized statistic, which indicates the strength of trends for parameters to decrease or increase across the groups. Logistic regression was employed to assess the relationships of %FM or %MMI with moderate-to-severe CKD. All the models were adjusted for potential confounders that are known or suspected to influence the relationship of fat and muscle mass with CKD in older adults. Model 1 was adjusted for age and sex; Model 2 was adjusted for the model 1 parameters plus BMI; Model 3 was adjusted for the Model 2 parameters plus the serum TC and triglyceride concentrations; and Model 4 was adjusted for the Model 3 parameters plus smoking and drinking. The results are presented as odds ratios (ORs) with 95% confidence intervals (CIs). During logistic regression analysis, the lowest quartile %FM and %MMI groups were used as the reference groups. SPSS software, version 20.0 (IBM, Inc., Armonk, NY, USA), was used for the statistical analyses. 

## 3. Results

### 3.1. Characteristics of the Study Participants and the Differences between Men and Women

Table 1 shows the characteristics of the study participants and the differences between men and women. There were no differences between men and women, except with respect to SBP and DBP (*p* < 0.05) and creatinine (*p* < 0.01).

### 3.2. Anthropometric Parameters

The study participants were categorized by eGFR as follows: (A) a normal group (eGFR ≥ 60 mL/min/1.73 m^2^, *n* = 102), (B) a mild CKD group (eGFR 45–59 mL/min/1.73 m^2^, *n* = 98), and (C) a moderate to severe CKD group (eGFR < 45 mL/min/1.73 m^2^, *n* = 36). Table 2 shows the anthropometric characteristics and trends adjusted for age and sex, and trends by eGFR categories. ANCOVA showed that the participants in group (A) were taller than those in groups (B) and (C), but there was no difference between groups (B) and (C). The body weight of group (A) was higher than that of group (C), but there were no differences between groups (A) and (B) or (B) and (C). There were trends for height (standardized statistic (SS) = −6.40, *p* < 0.01) and weight (SS = −2.48, *p* < 0.05) to decrease and for age to increase (SS = 2.54, *p* < 0.05) across groups (A) to (C). There were no differences among the three groups or trends with respect to BMI.

### 3.3. Body Composition Parameters

Table 3 shows characteristics of body composition adjusted for age and sex, and trends by eGFR categories. To avoid the confounding effects of age and sex, ANCOVA was performed, using age and sex as covariates. Post-hoc and trend tests demonstrated differences among the three groups for all of the variables. LM, ASM, MMI, and %MMI did not differ between groups (B) and (C), but were higher in group A than in the other groups. The %FM in groups (B) and (C) was higher than that of group A, but there were no differences between groups (B) and (C). With the exception of %FM, the trend test also demonstrated decreases across groups (A) to (C) (SS = −5.51, −5.60, −3.95, and −5.47, respectively; *p* < 0.01 for all). The opposite tendency was identified for %FM (SS = 4.27, *p* < 0.01).

### 3.4. Biochemical Parameters

The biochemical measurements and trends by eGFR categories are presented in Table 4. Multiple comparisons demonstrated differences among the three groups for all of the variables, except SBP and plasma insulin. The DBP of group (B) was higher than that of group (C). Creatinine concentration increased (SS = 12.66, *p* < 0.01) and eGFR decreased (SS = −15.45, *p* < 0.01) across groups (A) to (C). Although there was no significant difference in TC or HDLC between groups (A) and (B) or (B) and (C), they significantly differed between groups (A) and (C) (*p* < 0.05). In addition, there were trends for TC (SS = −2.58, *p* < 0.05) and HDLC (SS = −2.64, *p* < 0.01) to decrease across groups (A) to (C). TG concentration tended to increase across groups A to C (SS = 3.15, *p* < 0.01) and there was a significant difference between the (A) and (C) groups (*p* < 0.05). FPG did not differ between groups A and B, but the values for both these groups were lower than that for group (C) (*p* < 0.01). Finally, there was a significant increasing trend in plasma insulin concentration (SS = 2.43, *p* < 0.05).

### 3.5. Odds Ratios for the Percentage of Fat Mass

**Figure 2:**
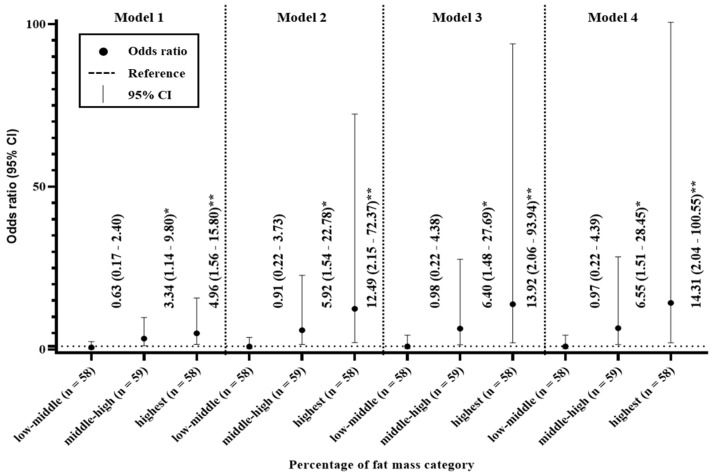
Odds ratios for the relationships between the percentage of fat mass and chronic kidney disease. Model 1 was adjusted for age and sex; Model 2 was adjusted for the model 1 parameters plus BMI; Model 3 was adjusted for the Model 2 parameters plus the serum total cholesterol and triglyceride concentrations; and Model 4 was adjusted for the Model 3 parameters plus smoking and drinking * *p* < 0.05. ** *p* < 0.01.

The relationship of %FM with moderate-to-severe CKD is shown in Figure 2. When the participants were divided into quartiles on the basis of %FM, high %FM were more likely to have moderate-to-severe CKD. In Model 1, the middle-high and highest quartile had odds ratios of 3.34 (95% CI 1.14–9.80) and 4.96 (95% CI 1.56–15.80), respectively, of moderate-to-severe CKD, compared with the lowest quartile. Similarly, in Models 2–4, the middle-high and highest quartile had odds ratios of 5.92 (95% CI 1.54–22.78) and 12.49 (95% CI 2.15–72.37); 6.40 (95% CI 1.48–27.69) and 13.92 (95% CI 2.06–93.94); and 6.55 (95% CI 1.51–28.45) and 14.31 (95% CI 2.04–100.55), respectively, of moderate-to-severe CKD, compared with the lowest quartile.

### 3.6. Odds Ratios for the Percentage of Muscle Mass Index

**Figure 3:**
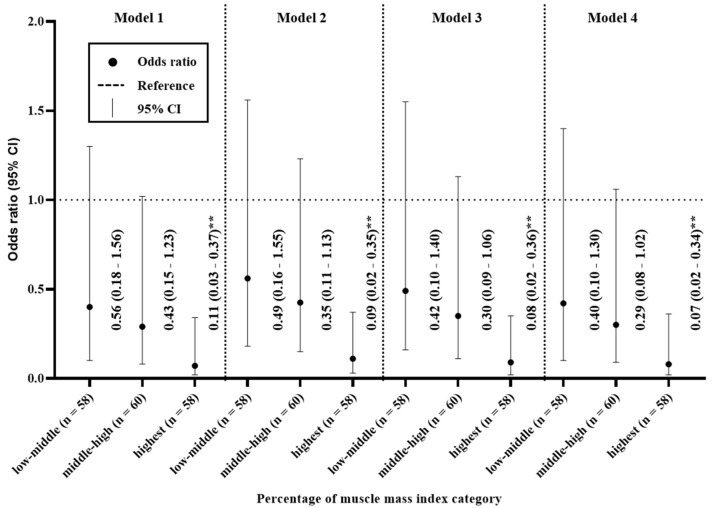
Odds ratios for the relationships between percentage muscle mass index and chronic kidney disease. Model 1 was adjusted for age and sex; Model 2 was adjusted for the model 1 parameters plus BMI; Model 3 was adjusted for the Model 2 parameters plus the serum total cholesterol and triglyceride concentrations; and Model 4 was adjusted for the Model 3 parameters plus smoking and drinking. ** *p* < 0.01.

Figure 3 shows the relationship between %MMI and moderate-to-severe CKD. When the participants were divided into quartiles on the basis of their %MMI, individuals with low %MMI were more likely to have moderate-to-severe CKD. In Model 1, the highest quartile had an odds ratio of 0.11 (95% CI 0.03–0.37) for moderate-to-severe CKD, compared with the lowest quartile. Similarly, for Models 2–4, the highest quartile had odds ratios of 0.09 (95%CI 0.02–0.35), 0.08 (95%CI 0.02–0.36), and 0.07 (95%CI 0.02–0.34), respectively, for moderate-to-severe CKD, compared with the lowest quartile.

## 4. Discussion

We conducted a cross-sectional pilot study to assess the relationships of fat and muscle mass with CKD in older adults. Our principal findings were as follows. First, we identified trends for age to increase and weight to decrease with CKD, but no trend with respect to BMI (Table 2). Second, %FM was higher and %MMI was lower in participants with moderate to severe CKD versus those without kidney disease (Table 3). Third, circulating TG and insulin concentrations increased with CKD, and FPG was higher in participants with moderate to severe CKD than in those without kidney disease or mild CKD (Table 4). Finally, the participants in the middle-high, and highest quartile for %FM were 6.55 and 14.31 times more likely to have moderate to severe CKD. Conversely, the participants in the highest quartile for %MMI were 0.07 times less likely to have moderate to severe CKD (Figure 2 and Figure 3). These findings suggest that a body composition that includes high FM and low muscle mass may be more strongly associated with CKD than obesity per se.

It has long been known that aging and obesity are associated with CKD [1,7,17,18]. The findings of the present study are consistent with those of studies that have shown CKD to be related to aging, but not with those indicating that obesity, assessed using BMI, is closely related to CKD (Table 1). Recently, the measurement of fat accumulation in either lean or obese individuals has been shown to identify individuals at risk of several medical conditions more effectively than a BMI-based definition of obesity [11]. In the case of CKD, Kim et al. (2014) showed that high body fat is a significant risk factor for CKD, regardless of body weight [19]. In addition, Chen et al. (2018) showed a detrimental effect of high body fat on kidney function [20]. In the present study, %FM, along with the circulating concentrations of TG, FPG, and insulin, tended to increase with CKD (Table 2 and Table 3). Consistent with these findings, previous studies have shown that fat accumulation is closely related to the onset of proteinuria, which may be indicative of detrimental effects of fat accumulation on kidney hemodynamics [21]. Such a relationship is supported by evidence of crosstalk between adipose and blood vessels [22,23]. The adipose tissue of individuals with obesity becomes highly inflamed and causes vascular dysfunction through greater secretion of vasoconstrictors, including via the renin-angiotensin-aldosterone system, superoxide, and proinflammatory adipokines, which are important contributors to endothelial activation, vascular inflammation, and neointimal formation [24]. Consequently, it is thought that fat accumulation is closely associated with CKD. Furthermore, logistic regression analysis of the present data showed that the middle-high quartile had odds ratios of 3.34–6.55 for moderate-to-severe CKD in Models 1–4, relative to the lowest quartile; and the highest quartile had odds ratios of 4.96–14.31 for moderate-to-severe CKD in these models (Figure 2). 

Interestingly, all of the muscle mass-related variables (LM, ASM, MMI, and %MMI) tended to decrease with increasing severity of CKD (Table 2), and the highest quartile had odds ratios of 0.11–0.07 for moderate-to-severe CKD, compared with the lowest quartile, in Models 1–4 (Figure 3). Many recent studies have shown the importance of the maintenance of muscle mass in older adults, which emphasizes the need to minimize the loss of muscle mass with age [25,26]. Because excessive muscle mass loss in older adults can cause several problems, such as frailty and sarcopenia [10,25,26], it is essential to investigate the relationship between the loss of muscle mass and CKD in older adults. However, it is still uncertain whether a loss of muscle mass is associated with CKD, or whether CKD is a cause of a loss of muscle mass. Workeneh and Mitch (2010) reported a possible mechanism for the link between the loss of muscle mass and CKD as follows: inflammation, metabolic acidosis, and angiotensin II cause defects in insulin/ insulin-like growth factor 1 (IGF-1)-activated intracellular signaling, which aggravates muscle protein degradation by the ubiquitin proteasome system and caspase-3 [27]. Thus, excessive fat accumulation may be associated with CKD, which accelerates the loss of muscle mass in older adults. This implies that, to prevent rapid kidney disease and muscle mass loss in older adults, FM should be reduced.

The limitations of the present study included its small sample size, the lack of adjustment for confounding factors such as lifestyle characteristics, the lack of a sex-based analysis, and the lack of identification of a precise mechanism. Because the small sample size limited the statistical power of the present study, a larger-scale study should be conducted in the future. Recent epidemiological studies have revealed associations between lifestyle characteristics, such as physical activity and dietary habits, and a variety of health problems. These findings imply that the relationships between high fat and low muscle masses and CKD could be rendered more accurate by adjusting the data for variations in lifestyle characteristics. There is a difference in body composition between men and women: men have a higher mean muscle mass and a lower mean FM than women. Thus, high %FM mass and low %MMI may not confer the same risks of the moderate to severe CKD in each sex, and a sex-specific analysis should be conducted in a future study. The mechanism for the harmful effects of fat accumulation on kidney function has been investigated for decades, but it is still unclear why CKD is associated with a loss of muscle mass. In the future, the mechanism involved should be determined in detail.

## 5. Conclusions

%FM increases and %MMI decreases with the severity of CKD, regardless of BMI. The participants in the middle-high, and highest quartile for %FM were 6.55 and 14.31 times more likely to have moderate to severe CKD. Conversely, the participants in the highest quartile for %MMI was 0.07 times less likely to have moderate to severe CKD. The results of this cross-sectional pilot study suggest that a body composition that includes high FM and low muscle mass may be more strongly associated with CKD than obesity per se.

## Figures and Tables

**Table 1:** Characteristics of the study participants and the differences between men and women.

	Total (*n* = 236)	Men (*n* = 47)	Women (*n* = 189)	*p* Value
Age, year	76.8 ± 3.7	77.3 ± 3.7	76.7 ± 3.7	=0.25
Height, cm	155.7 ± 6.8	155.5 ± 6.0	155.7 ± 7.0	=0.83
Body weight, kg	60.0 ± 8.7	58.2 ± 8.4	60.4 ± 8.7	=0.11
BMI, kg/m^2^	24.8 ± 3.2	24.1 ± 3.2	24.9 ± 3.1	=0.09
LM, kg	37.2 ± 5.7	36.1 ± 5.3	37.5 ± 5.8	=0.13
ASM, kg	15.4 ± 3.2	15.2 ± 3.7	15.5 ± 3.0	=0.55
MMI, kg/m^2^	6.3 ± 1.0	6.3 ± 1.5	6.3 ± 0.8	=0.65
%MMI, %	25.8 ± 4.5	26.3 ± 7.0	25.6 ± 3.6	=0.37
%FM, %	37.5 ± 7.7	37.5 ± 7.6	37.5 ± 7.7	=0.96
SBP, mm Hg	139.1 ± 18.3	145.0 ± 17.8	137.7 ± 18.2	<0.05
DBP, mm Hg	74.3 ± 11.3	78.3 ± 12.4	73.3 ± 10.8	<0.05
CRE, mg/dL	0.8 ± 0.2	1.0 ± 0.2	0.8 ± 0.2	<0.01
eGFR, mg/dL	57.7 ± 12.4	58.9 ± 11.8	57.4 ± 12.6	=0.44
TC, mg/dL	184.3 ± 36.3	176.5 ± 34.2	186.3 ± 36.6	=0.10
HDLC, mg/dL	53.7 ± 13.7	50.3 ± 13.0	54.5 ± 13.8	=0.06
TG, mg/dL	131.2 ± 61.5	125.8 ± 53.1	132.5 ± 63.5	=0.51
FPG, mg/dL	99.6 ± 23.2	102.7 ± 19.8	98.8 ± 23.9	=0.31
Insulin, IU	8.8 ± 7.7	8.5 ± 7.2	8.9 ± 7.8	=0.77
Smoking (yes)	5.1 % (12)	4.3 % (2)	5.3 % (10)	=0.77
Alcohol (yes)	25.0 % (59)	19.1 % (9)	26.5 % (50)	=0.30

Values are means ± standard deviation or % (number of cases). BMI = body mass index; LM = whole body lean mass; ASM = appendicular skeletal muscle mass; MMI = muscle mass index; % MMI = percentage of muscle mass index; % fat mass = percentage of whole body fat mass; SBP = systolic blood pressure; DBP = diastolic blood pressure; CRE = Creatinine; TC = total cholesterol; HDLC = high density lipoprotein-cholesterol; TG = triglyceride; FPG fasting plasma glucose.

**Table 2:** Anthropometric characteristics, and trends, adjusted for age and sex, by eGFR categories.

	eGFR Category (mL/min/1.73 m^2^)			Post Hoc	^b^ SS	*p* for ^b^ Trend
	(A) eGFR ≥ 60	(B) eGFR 45–59.9	(C) eGFR < 45			
	(*n* = 102)	(*n* = 98)	(*n* = 36)			
^#^ Age, year	76.1 (75.4, 76.8)	77.2 (76.5, 77.9)	77.6 (76.3, 78.3)	NS	2.54	<0.05
^a^ Height, cm	159.0 (157.8, 160.2)	153.7 (152.4,154.9)	151.5 (149.5, 153.6)	A > B, C	−6.40	<0.01
Body weight, kg	61.6 (59.9, 63.3)	59.2 (57.5, 60.9)	57.6 (54.7, 60.4)	A > C	−2.48	<0.05
BMI, kg/m^2^	24.5 (23.7, 25.0)	25.0 (24.4, 25.6)	25.1 (24.1, 26.1)	NS	1.53	=0.13

Values are means and 95% CI. ^#^ One-way analysis of variance was employed to adopted to assess the difference among the groups ^a^ Kruskal-Wallis test was applied to assess the difference among three groups. ^b^ Jonckheere-Terpstra test was used to assess the trend by eGFR categories. eGFR = estimated glomerular filtration rate; SS = standardized statistic; NS = not significant; BMI = body mass index.

**Table 3:** Body composition parameters, adjusted for age and sex, and trends by eGFR categories.

	eGFR Category (mL/min/1.73 m^2^)			Post Hoc	^b^ SS	*p* for ^b^ Trend
	(A) eGFR ≥ 60	(B) eGFR 45–59.9	(C) eGFR < 45			
	(*n* = 102)	(*n* = 98)	(*n* = 36)			
LM, kg	39.8 (38.8, 40.9)	35.4 (34.4, 36.4)	34.6 (32.9, 36.3)	A > B, C	−5.51	<0.01
ASM, kg	16.8 (16.2, 17.3)	14.6 (14.0, 15.2)	13.8 (12.8, 14.7)	A > B, C	−5.60	<0.01
MMI, kg/m^2^	6.6 (6.4, 6.8)	6.2 (6.0, 6.4)	6.0 (5.7, 6.3)	A > B, C	−3.95	<0.01
%MMI, %	27.2 (26.3, 28.0)	24.9 (24.0, 25.8)	24.0 (22.6, 25.5)	A > B, C	−5.47	<0.01
%FM, %	35.0 (33.6, 36.5)	39.5 (38.0, 41.0)	39.3 (36.8, 41.7)	A < B, C	4.27	<0.01

Values are means and 95% CI. ^b^ Jonckheere-Terpstra test was used to assess the trend by eGFR categories. eGFR = estimated glomerular filtration rate; SS = standardized statistic; NS = not significant; LM = whole body lean mass; BMI = body mass index; ASM = appendicular skeletal muscle mass; MMI = muscle mass index; %MMI = percentage of muscle mass index; %FM = percentage of whole body fat mass.

**Table 4:** Biochemical characteristics, adjusted for age and sex, and trends by eGFR categories.

	eGFR Category (mL/min/1.73 m^2^)			Post Hoc	^b^ SS	*p* for ^b^ Trend
	(A) eGFR ≥ 60	(B) eGFR 45-59.9	(C) eGFR < 45			
	(*n* = 102)	(*n* = 98)	(*n* = 36)			
SBP, mm Hg	138.0 (134.4, 141.5)	141.7 (138.1, 145.3)	135.5 (129.5, 141.4)	NS	0.48	=0.63
DBP, mm Hg	73.3 (71.4, 75.8)	76.3 (74.1, 78.5)	70.9 (67.2, 74.5)	B > C	−0.61	=0.54
CRE, mg/dL	0.7 (0.65, 0.69)	0.8 (0.8, 0.9)	1.1 (1.1, 1.2)	A < B < C	12.66	<0.01
eGFR, mg/dL	68.8 (67.7, 70.0)	53.2 (52.1, 54.3)	38.3 (36.4, 40.1)	A > B > C	−15.45	<0.01
TC, mg/dL	189.3 (182.3, 196.2)	183.8 (176.7, 190.9)	171.6 (159.9, 183.4)	A > C	−2.58	<0.05
HDLC, mg/dL	56.0 (53.3, 58.6)	53.3 (50.7, 56.0)	48.3 (43.8, 52.7)	A > C	−2.64	<0.01
TG, mg/dL	120.7 (108.7, 132.7)	135.2 (123.0, 147.4)	150.0 (129.9, 170.1)	A < C	3.15	<0.01
FPG, mg/dL	97.3 (92.9, 101.8)	97.1 (92.6, 101.6)	112.7 (105.2, 120.1)	A, B < C	1.96	=0.05
Insulin, IU	7.8 (6.2, 9.3)	9.7 (8.2, 11.2)	9.6 (7.0, 12.1)	NS	2.43	<0.05

Values are means and 95% CI. ^b^ Jonckheere-Terpstra test was used to assess the trend by eGFR categories. eGFR = estimated glomerular filtration rate; SS = standardized statistic; NS = not significant; SBP = systolic blood pressure; DBP = diastolic blood pressure; CRE = Creatinine; TC = total cholesterol; HDLC = high density lipoprotein-cholesterol; TG = triglyceride; FPG fasting plasma glucose.

## Supplementary Materials

The following are available online at https://www.mdpi.com/1660-4601/17/23/9124/s1, Table S1: Average values of eGFR and body composition parameters according to body composition quartile.
