# Supplementary material for: Relationships of Fat and Muscle Mass with Chronic Kidney Disease in Older Adults: A Cross-Sectional Pilot Study
Source: Int J Environ Res Public Health. 2020 Dec 7;17(23):9124. doi: 10.3390/ijerph17239124 (PMC7731281; doi:10.3390/ijerph17239124)
Supplement: Supplementary file 1 [file ijerph-17-09124-s001.pdf]

Supplementary

Table S1. Average values of eGFR and body composition parameters according to body composition quartile.

	LM	
	Average value of LM (kg)	Average value of eGFR (mg/dL)
The lowest quartile (n =70)	32.5	54.4
The low-middle quartile (n = 52)	35.0	55.0
The middle-high quartile (n = 56)	37.7	55.0
The highest quartile (n = 58)	44.3	66.7
	**ASM**	
	Average value of ASM (kg)	Average value of eGFR (mg/dL)
The lowest quartile (n = 64)	12.2	53.5
The low-middle quartile (n = 54)	14.2	53.8
The middle-high quartile (n = 60)	15.6	54.8
The highest quartile (n = 58)	19.8	68.9
	**MMI**	
	Average value of MMI (kg/m^2)	Average value of eGFR (mg/dL)
The lowest quartile (n = 64)	5.5	53.5
The low-middle quartile (n =54)	6.1	53.8
The middle-high quartile (n = 60)	6.4	54.8
The highest quartile (n =58)	7.4	68.9
	**%MMI**	
	Average value of %MMI (%)	Average value of eGFR (mg/dL)
The lowest quartile (n = 58)	21.7	52.7
The low-middle quartile (n = 60)	24.0	55.4
The middle-high quartile (n =58)	26.2	55.4
The highest quartile (n = 60)	31.1	67.4
	**%FM**	
	Average value of %FM (%)	Average value of eGFR (mg/dL)
The lowest quartile (n = 58)	27.7	62.6
The low-middle quartile (n = 59)	35.9	60.8
The middle-high quartile (n = 58)	40.6	53.7
The highest quartile (n = 61)	46.7	53.5
